# Longitudinal trajectories of apathy in Huntington’s disease: a 6-year follow-up study

**DOI:** 10.1007/s00415-026-13825-x

**Published:** 2026-04-21

**Authors:** Emilie Poulsen, Rebecca K. Hendel, Birna Ásbjörnsdóttir, Lena E. Hjermind, Jørgen E. Nielsen, Asmus Vogel

**Affiliations:** 1 Danish Dementia Research Centre, Department of Neurology, Copenhagen University Hospital, Rigshospitalet, Copenhagen, Denmark; 2 Department of Psychology, University of Copenhagen, Copenhagen, Denmark; 3 Department of Clinical Medicine, University of Copenhagen, Copenhagen, Denmark

**Keywords:** Huntington’s disease, Apathy, Longitudinal study, Neuropsychiatric symptoms

## Abstract

**Objective:**

Apathy is a common and debilitating neuropsychiatric symptom in Huntington’s disease (HD), yet its long-term trajectory remains poorly characterized. This study examined changes in apathy in HD gene expansion carriers (HDGECs) over 6 years, using a multidimensional measure, and investigated associations with cognition, motor symptoms and depression.

**Methods:**

Eighty-two HDGECs (premanifest and manifest) completed assessments at Time 0 and Time 1 with a mean follow-up interval of 6 years. Apathy was measured using the Lille Apathy Rating Scale (LARS) and the Problem Behaviors Assessment–short (PBA-s). Depressive symptoms were assessed with the Hamilton Depression Rating Scale, while social cognition and executive functioning were measured using the Emotion Hexagon and Symbol Digit Modalities Test. Within-person changes were examined using paired statistical tests and associations with clinical variables were evaluated using correlation analyses.

**Results:**

Total apathy scores increased significantly over 6 years, with small changes (LARS: 1.38 points; PBA-s: 1.11 points). Premanifest participants showed a selective decline in Action Initiation, whereas manifest participants exhibited a broader worsening of total apathy. Individual trajectories were variable, with both worsening and improvement observed. An increase in depressive symptoms was significantly correlated with changes in apathy but accounted for little variance.

**Conclusion:**

Apathy in HD shows a small but significant increase over 6 years, characterized by marked heterogeneity and changes in Action Initiation. The limited association with depression and cognition highlights apathy as an independent neuropsychiatric feature. These findings underscore the heterogeneous nature of apathy progression and the value of multidimensional assessment in longitudinal studies.

## Introduction

Huntington’s disease (HD) is a progressive neurodegenerative disorder caused by an expanded cytosine–adenine–guanine (CAG) trinucleotide repeat in the *HTT* gene on chromosome 4, which encodes the huntingtin protein [1]. The mutant huntingtin protein exerts toxic effects on neurons, leading to widespread dysfunction and cell death. This pathology manifests clinically as a combination of motor, cognitive, and psychiatric symptoms [1, 2].

Traditionally, HD diagnosis relies on the presence of motor symptoms combined with a confirmatory genetic test or family history [3]. However, accumulating evidence indicates that cognitive and psychiatric changes often precede the clinical diagnosis of motor onset, which is based on the presence of sufficient motor signs, by several years. These early symptoms include executive dysfunction and neuropsychiatric disturbances such as apathy [4, 5].

Apathy is consistently reported as one of the most prevalent symptoms in HD and is considered highly distressing by individuals with HD and their caregivers [3, 6]. Prevalence estimates range from 50 to 99% across disease stages [7, 8]. It substantially reduces quality of life, as individuals disengage from daily activities while caregivers experience increased burden [9]. Furthermore, apathy has emerged as a potential indicator of disease progression, with studies suggesting its presence in premanifest HD and gradual worsening over time [5, 10, 11].

Despite its clinical importance, the conceptualization of apathy remains inconsistent. It is commonly defined as a reduction in goal-directed behavior [12], yet researchers debate whether it reflects diminished interest and motivation or a multidimensional construct encompassing emotional, cognitive, and behavioral components [5, 13]. Evidence suggests that apathy comprises distinct subtypes, and some studies indicate that only certain dimensions of apathy may be affected in HD. Previous work has reported changes particularly in subtypes related to cognitive apathy and reduced action initiation, while emotional apathy has been less consistently reported [14, 15].

Longitudinal research on apathy in HD is limited. Existing studies typically span 1 to 2 years [14, 16–20] while data from the multinational TRACK-HD, Enroll-HD and Cooperative Huntington Observational Research Trial (COHORT) studies have contributed to studies spanning up to 5 years [10, 11, 21, 22]. Findings generally suggest that the prevalence and severity of apathy increase with advancing HD [5, 8, 22, 23]. Yet, clinical cohorts show a mixed trajectory with a gradual rise over years in some samples, but relative stability over shorter (18–24 months) periods in some groups [8, 18, 20, 22]. Additionally, few longitudinal studies have explicitly assessed apathy progression in HD, with multiple studies instead using longitudinal data primarily to assess associations between apathy and other clinical variables [10, 11].

Most studies rely on brief clinician-rated measures such as the Short Problem Behaviors Assessment for HD (PBA-s), which is rated for frequency and severity among a set of neuropsychiatric symptoms [24]. The PBA-s assesses apathy through a single domain that mainly reflects lack of initiative, and while this approach captures a clinically relevant aspect of apathy, it provides limited information about the multidimensional nature of apathy or its underlying mechanisms [8, 11].

In contrast, studies that have used more comprehensive apathy measures have typically had shorter follow-up periods [8, 18] or have largely focused on manifest HD gene expansion carriers (HDGECs) with limited inclusion of premanifest participants [8, 22].

The present study addresses a gap in the literature, as long-term (> 5 years) longitudinal data on apathy progression in HDGECs using multidimensional measures are currently lacking. We previously examined apathy in a cohort of premanifest and manifest HDGECs and family controls using the Lille Apathy Rating Scale (LARS) [15]. Building on these findings, the aim of this study was to investigate the 6-year trajectory of apathy in this broad cohort using a multidimensional apathy scale. A secondary aim was to identify clinical predictors (cognitive, behavioral and affective measures) that may be associated with changes in apathy. Based on previous evidence that apathy increases with advancing HD, the assumption was that total apathy would worsen over a 6-year period.

## Methods

### Participants

#### Time 0

The baseline cohort at Time 0 consisted of 82 HDGECs enrolled between 2018 and 2019 through the Neurogenetics Clinic at the Danish Dementia Research Centre, Rigshospitalet, Denmark [15, 25]. Thirty-two previously at-risk offspring of HDGECs who tested negative for the HD gene expansion served as family controls to account for shared social and environmental factors.

HDGECs were eligible for inclusion if they had a CAG repeat length of ≥ 39, a Unified Huntington’s Disease Rating Scale–99 total motor score (UHDRS-TMS) [26] of ≤ 35, a Mini-Mental State Examination (MMSE) [27] score of ≥ 24, and a Montreal Cognitive Assessment (MoCA) [28] score of ≥ 19. Based on motor symptom severity, HDGECs were classified as either manifest or premanifest. Participants with a UHDRS-TMS score > 5 were categorized as manifest HDGECs (*N* = 42), while those scoring ≤ 5, indicating no substantial motor signs, were classified as premanifest HDGECs (*N* = 40). This cutoff was consistent with criteria previously applied in our cohort study [29].

Exclusion criteria included the presence of other neurological disorders and ongoing alcohol or drug abuse. Participants were also excluded if they were unable to complete assessments due to insufficient Danish language proficiency. All participants had received genetic counseling and knew their genetic status before study participation.

The study protocol was approved by the Ethics Committee of the Capital Region of Denmark (H-17002606) and adhered to the Declaration of Helsinki and its subsequent amendments. Written informed consent was obtained from all participants prior to inclusion.

#### Time 1

Participants at Time 1 included HDGECs and healthy family controls (HC) who had previously taken part in the initial assessment (Time 0) and consented to participate in the follow-up examination conducted in 2024–2025. All individuals from the original cohort were contacted and invited to take part in the follow-up, of which 55 HDGECs and 27 HC participated.

Participants were reclassified as either premanifest or manifest HDGECs based on their UHDRS-TMS, using the same criteria applied at Time 0. The same assessments were administered at both time points to ensure consistency in data collection.

### Assessments

All participants underwent neurological, neuropsychiatric, and neuropsychological assessments. The same neuropsychologist conducted all neuropsychological examinations at Time 0 (RKH) and Time 1 (EP) to ensure consistency, while certified Enroll-HD neurologists at our clinic performed the neurological examinations.

Motor symptoms were assessed using the UHDRS-TMS [26], while functional capacity was assessed using the UHDRS-Total Functional Capacity (TFC) scale [26]. The UHDRS-TFC comprises five clinician-rated items covering work, finances, domestic responsibilities, activities of daily living, and level of care. Scores range from 0 to 13 with higher scores indicating better functional capacity [26].

Depressive symptoms were assessed with the Hamilton Depression Rating Scale (HAM-17) [30], a semi-structured interview covering 17 symptoms related to depression.

### Apathy

Apathy was assessed using the LARS [31].

The LARS is a 33-item standardized structured interview covering nine domains of apathy. The first three items are rated on a 5-point Likert scale (− 2 to + 2). Items 4–33 consist of positively worded yes/no questions coded as − 1 or + 1, with an additional “not applicable” option (0) when relevant. A global score ranging from − 36 to + 36 was computed, with higher values indicating greater apathy. In addition, four apathy subscale scores (Intellectual Curiosity, Emotion, Action Initiation, and Self-Awareness) were calculated by averaging the items within each subtype (range − 4 to + 4).

Behavioral symptoms were assessed with the PBA-s [24], an 11-item semi-structured interview rating symptom severity and frequency on 5-point scales based on patient, informant, and clinician observation. The PBA-s includes single items assessing apathy and depression derived from the original PBA-HD [32]. In addition to the apathy item, the clinician-rated depression item was included in exploratory analyses to examine the relationship between apathy and depressive symptoms and to assess potential overlap between apathy and depression measures.

### Neuropsychological assessment

Participants completed screening measures including the MMSE and MOCA [27, 28] to check for cognitive deficits. The neuropsychological assessment included a wide range of tests. This report focuses specifically on data relevant to the evaluation of apathy in individuals with HD, and only two tests are reported.

Executive functioning and processing speed were assessed using the Symbol Digit Modalities Test (SDMT) in which participants must match symbols to corresponding numbers according to a key within a fixed time limit [33]. Social cognition was evaluated with the Emotion Hexagon (EH) test [34], which consists of 30 images depicting morphed facial expressions of the six basic emotions: happiness, surprise, fear, sadness, anger, and disgust. SDMT and EH have previously been described in detail in Hendel et al. [35].

### Statistical analysis

All analyses were conducted using SPSS version 29 and R Studio version 4.5.0.

Normality was evaluated using the Shapiro–Wilk test and homogeneity of variance with Levene’s test. Group differences in demographic and clinical variables at Time 0 and Time 1 were examined using one-way ANOVA for normally distributed data, or the Kruskal–Wallis test for non-normal data. When comparisons between two groups were required (HDGECs vs. HC, and premanifest vs. manifest HDGECs), independent-samples *t* tests or Mann–Whitney U tests were used based on normality. For subgroup comparisons, participants were grouped according to their classification at Time 0. This baseline classification was used for all subgroup comparisons and longitudinal analyses. Descriptive characteristics at Time 1 were presented according to disease stage at Time 1 for descriptive purposes only.

Within-person change from Time 0 to Time 1 was assessed using paired-samples *t* tests for normally distributed variables and Wilcoxon signed-rank tests for non-normal variables.

To quantify change over time, delta(Δ) scores were computed for LARS Global, its subscales, the PBA-s Apathy score, and all clinical and cognitive measures (UHDRS-TMS, MoCA, MMSE, HAM-17, SDMT, and EH), defined as Time 1 minus Time 0.

To examine predictors of apathy progression, associations between changes in apathy and changes in clinical variables were assessed using Pearson’s *r* or Spearman’s *rho*, depending on the normality of distributions.

Statistical significance was set at *p* < 0.05. All tests were two-tailed unless a directional hypothesis was specified a priori. Based on prior findings suggesting progressive increases in apathy in HD, a directional hypothesis was specified that apathy would worsen over time, and this was tested using a one-tailed paired-samples t-test. To account for multiple comparisons, Bonferroni–Holm correction was applied separately to primary (LARS Global, PBA-s Apathy) and secondary (LARS subscales) outcomes. Adjusted p-values are reported only where multiple comparison correction altered the significance of the result.

## Results

### Participants

**Fig. 1:**
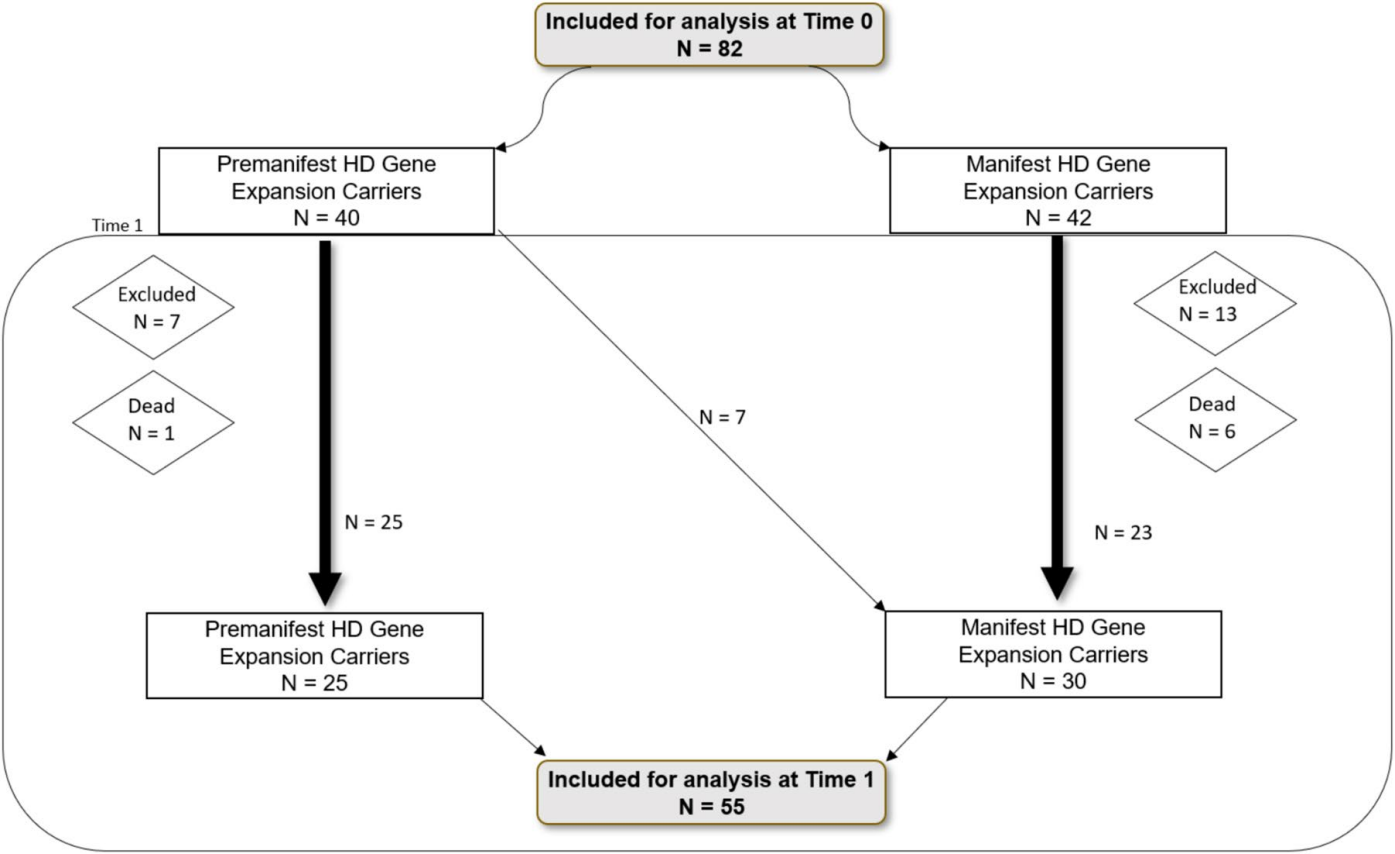
Diagram of participants included for analysis and categorized as premanifest or manifest HD gene expansion carriers at Time 0 and Time 1 *HD*Huntington’s disease, *N* total number of participants

The mean follow-up interval between Time 0 and Time 1 was 6 years (M = 2203 days). Of the 82 HDGECs assessed at Time 0, a total of 55 HDGECs (25 premanifest, 30 manifest) completed the follow-up evaluation at Time 1, while 27 were not reassessed (see Fig. 1). Of the 27 lost to follow-up, seven participants had passed away (1 premanifest, 6 manifest), seven manifest HDGECs were unable to participate, and thirteen individuals (7 premanifest, 6 manifest) declined or could not be reached. Seven HDGECs transitioned from premanifest to manifest status between assessments. Of the 32 HC assessed at Time 0, five were lost to follow-up, resulting in 27 HC reassessed at Time 1.

When comparing HDGECs who completed the assessment at Time 1 (*n* = 55) with those lost to follow-up (*n* = 27), significant baseline differences emerged. Participants lost to follow-up had lower cognitive performance (MoCA: *U* = 466.5, *p* = 0.006; MMSE: *U* = 502, *p* = 0.015), worse motor function (UHDRS-TMS: *U* = 383.0, *p* = 0.001), and higher disease burden (CAP score: *U* = 448.0, *p* = 0.004). In contrast, baseline apathy (LARS Global) and depressive symptoms (HAM-17) did not differ significantly between groups.

### Background characteristics and apathy at Time 0 and Time 1

Table 1 presents background characteristics for the HDGECs and HC at Time 0 and Time 1, including their results on cognitive screening, motor, depressive symptoms and apathy measures.

**Table 1:** Background characteristics and scores on total apathy measures for the HD gene expansion carriers at Time 0 and Time 1, and the controls

	HD Gene Expansion Carriers Time 0			HD Gene Expansion Carriers Time 1		
	Premanifest	Manifest	HC	Premanifest	Manifest	HC
N	40	42	32	25	30	27
Age (years)	41.3 (11.0)	52.5 (12.3)^‡^	48.1 (14.1)	45.5 (10.1)*	56.7 (12.2)^‡^	53.7 (13.7)
Sex (M/F)	24/16	22/20	13/19	16/9	18/12	10/7
CAG-repeat length	41.8 (2.4)	42.7 (2.5)	–	41.2 (2.2)	42.4 (2.3)	–
CAP Score	248.7 (80.3)	361.1 (92.3)^‡^	–	250.0 (80.4)	375.8 (94.9)^‡^	–
UHDRS-TMS	1.8 (1.7)*	30.3 (18.7)* ^‡^	1.3 (1.3)	1.8 (1.4)*	25.7 (14.9)* ^‡^	0.7 (1.4)
MoCA Score	28.1 (2.0)*	25.4 (2.9)* ^‡^	29.2 (1.2)	27.7 (2.1)	24.0 (3.5)* ^‡^	28.0 (1.9)
MMSE Score	28.8 (1.1)*	27.4 (1.9)* ^‡^	29.3 (1.1)	29.3 (0.8)	26.3 (3.2)* ^‡^	29.6 (0.9)
LARS Global Score	−25.2 (4.3)	−21.2 (6.7)* ^‡^	−26.3 (3.0)	−25.6 (4.0)	−19.7 (7.5)* ^‡^	−26.8 (2.7)
PBA-s Apathy Score^1^	0.1 (0.2)	1.2 (1.9)* ^‡^	0.1 (0.3)	0.3 (0.8)	2.7 (3.4)^*^^‡^	0.0 (0.2)
HAM-17 Score	2.5 (3.1)	4.2 (3.6)^‡^	2.7 (3.0)	2.6 (2.0)	4.1 (2.8)^‡^	2.6 (1.7)

Values are presented as mean ± SD unless noted otherwise. Group means at Time 1 are presented according to disease stage at Time 1; therefore, group composition differs from Time 0 due to disease progression in some participants

*HAM-17:* Hamilton Depression Rating Scale, *HC* Healthy controls, *HD* Huntington’s disease, *LARS* Lille Apathy Rating Scale, *MoCA* Montreal Cognitive Assessment, *N* total number of participants, *PBA-s* Problem Behaviors Assessment-short, *TMS* Total Motor Score, *UHDRS* Unified Huntington’s Disease Rating Scale

^1^Data missing from 4 HC at Time 1

*Statistically significant difference from the controls (*p* < 0.05)

^‡^Statistically significant difference from the premanifest HD (*p* < 0.05)

At Time 0, premanifest HDGECs had significantly higher UHDRS-TMS scores and lower cognitive screening scores than HC. By Time 1, premanifest HDGECs were significantly younger than HC and continued to show higher UHDRS-TMS scores, although their cognitive screening scores no longer differed from HC. The difference in age at Time 1 likely reflects changes in group composition over time, as participants with higher disease burden and worse motor and cognitive function were more likely to progress to manifest HD or be lost to follow-up, rather than dropout being driven by age itself.

As expected, manifest HDGECs exhibited significantly greater motor impairment and lower scores on cognitive screening measures than both premanifest participants and HC at both time points. Manifest HDGECs also reported more depressive symptoms (HAM-17) and greater apathy (LARS Global and PBA-s Apathy) than premanifest HDGECs at both time points.

### Changes in apathy over time in HDGECs

**Fig. 2:**
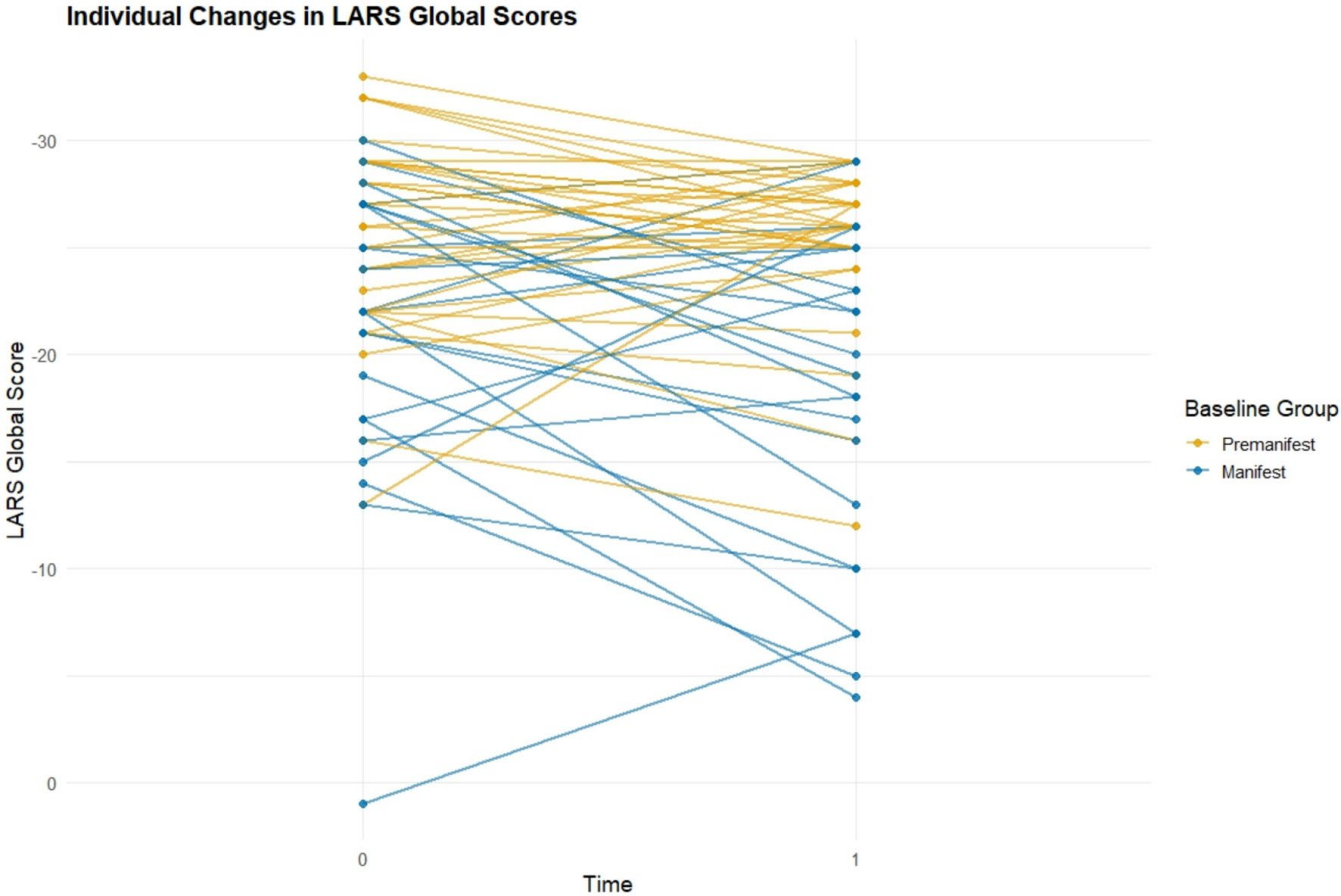
Spaghetti plot of individual trajectories of apathy (measured with the LARS Global Score) for Huntington’s gene expansion carriers from Time 0 to Time 1 *LARS* Lille Apathy Rating Scale

The trajectories of global apathy scores for HDGECs are illustrated in Fig. 2. Across HDGECs, apathy showed a modest overall increase over the 6-year follow-up. LARS Global scores worsened significantly from Time 0 to Time 1 (M = − 23.73 → − 22.35, t(54) = − 1.75, one-tailed *p* = 0.043, Cohen’s *d* = 0.24), indicating a small increase in apathy. The two-tailed test was not significant (*p* = 0.086). Individual trajectories of global apathy change in HDGECs are shown in Fig. 2. Clinician-rated apathy (PBA-s Apathy) also worsened significantly (M = 0.53 → 1.64, *Z* = − 3.27, *p* = 0.001), supporting an overall increase in apathy symptoms across the cohort.

To examine whether the LARS and PBA-s captured similar changes in apathy over time, correlations between change scores were calculated. Change in LARS Global scores was significantly correlated with change in PBA-s apathy scores (*r* = 0.363, *p* = 0.007), indicating that the two measures captured similar longitudinal changes in apathy.

Analysis of LARS subdomains showed that apathy related to Action Initiation worsened significantly (*M* = − 2.97 → − 2.40, t(54) = − 3.88, *p* < 0.001), while Emotional apathy (M = − 2.46 → − 2.77, t(54) = 2.03, *p* = 0.047) and reported self-awareness improved (M = − 2.16 → − 2.65, Z = − 2.00, *p* = 0.046). There were no changes in apathy related to Intellectual Curiosity. After Bonferroni–Holm correction across secondary outcomes, the improvements observed in Emotional apathy and Self-Awareness could not be considered significant.

### Changes in apathy over time in premanifest and manifest subgroups

In the premanifest subgroup, apathy also worsened on Action Initiation (M = − 3.48 → − 2.87, Z = − 3.57, *p* < 0.001). Scores on Self-Awareness improved (M = − 2.06 → − 2.90, Z = − 2.41, *p* = 0.016), while no significant changes were found for LARS Global, Intellectual Curiosity, or Emotion (all *p* > 0.12).

In the manifest subgroup, total apathy increased significantly over time, with LARS Global scores worsening from Time 0 to Time 1 (M = − 21.2 → − 18.0), t(22) = − 2.08, one-tailed *p* = 0.025, two-tailed *p* = 0.050, Cohen’s *d* = 0.43). No LARS subscales changed significantly (all *p* ≥ 0.11).

Clinician-rated apathy (PBA-s Apathy) worsened over time in both premanifest (M = 0.03 → 0.31, Z = − 2.11, *p* = 0.035) and manifest participants (M = 1.22 → 3.48, Z = − 2.83, *p* = 0.005), with higher scores at follow-up indicating greater apathy. Apathy scores for the premanifest and manifest HDGECs at Time 0 and Time 1 are presented in Table 2.

**Table 2:** Scores on total apathy measures for the premanifest and manifest HD gene expansion carriers at Time 0 and Time 1

	Premanifest		Manifest	
	Time 0	Time 1	Time 0	Time 1
N	32	32	23	23
Global LARS	− 25.6 (4.6)	− 25.5 (3.7)	− 21.2 (7.0)	− 18.0 (7.7)*
PBA-s Apathy subscale	0.03 (0.2)	0.3 (0.8)*	1.2 (2.0)	3.5 (3.6)*
LARS Intellectual Curiosity	− 2.8 (0.6)	− 2.7 (0.5)	− 2.4 (0.9)	− 2.1 (1.1)
LARS Emotions	− 2.6 (0.9)	− 2.9 (0.7)	− 2.2 (1.1)	− 2.6 (0.8)
LARS Action Initiation	− 3.5 (0.6)	− 2.9 (0.4)^*^	− 2.3 (1.2)	− 1.7 (1.3)
LARS Self-Awareness	− 2.1 (1.7)	− 2.9 (1.1)^*^	− 2.3 (1.5)	− 2.3 (1.3)

Values are presented as Mean ± SD. More positive/greater LARS scores demonstrate greater apathy. HDGECs are grouped according to their classification at Time 0

*HC* Healthy controls, *HD* Huntington’s disease, *HDGECs* Huntington’s disease gene expansion carriers, *LARS* Lille Apathy Rating Scale, *PBA-s* Problem Behaviors Assessment-short

*Within group change from Time 0 to Time 1 significant (*p* < 0.05)

Change scores were compared between premanifest and manifest participants. There were no statistically significant differences between groups in change in LARS Global scores, LARS subscales, or PBA-s apathy scores (all *p* > 0.05).

### Impact of motor symptoms, cognitive deficits and depression on changes in apathy

To examine predictors of apathy progression, we examined associations between changes in apathy (ΔLARS Global score) and changes in clinical variables using Spearman’s rho as most variables were non-normally distributed. Clinical variables included changes in motor symptoms (ΔUHDRS-TMS), changes in functional capacity (ΔUHDRS-TFC), changes in cognitive screening scores (ΔMoCA and ΔMMSE), changes in depressive symptoms (ΔHAM-17), changes in executive functioning (ΔSDMT), and changes in social cognition (ΔEH). Correlations between variables are shown in Table 3. Among all variables, only changes in depressive symptoms (ΔHAM-17) showed a small but significant association with changes in apathy (*r* = 0.30, *p* = 0.030), accounting for approximately 9% of the variance in apathy change. All other correlations were non-significant (*p*’s > 0.15).

**Table 3:**
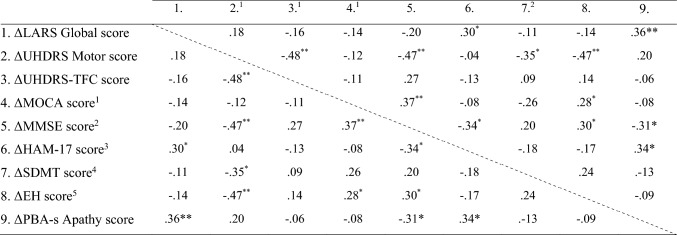
Correlation matrix assessing correlations between changes in global apathy and changes in other measures

Statistically significant at the level of *p* < 0.05. **Statistically significant at the level of *p* < 0.001

*EH* Emotion Hexagon, *HDGEC* Huntington’s disease gene expansion carrier, *HAM-17* Hamilton Depression Rating Scale, *LARS* Lille Apathy Rating Scale, *MOCA* Montreal Cognitive Assessment, *MMSE* Mini Mental State Examination, *PBA-s* Problem Behaviors Short Assessment, *SDMT* Symbol Digit Modality Test, *UHDRS* Unified Huntington’s Disease Rating Scale, *UHDRS-TFC* Unified Huntington’s Disease Rating Scale–Total Functional Capacity

^a^Data missing from one premanifest and three manifest HDGECs at Time 1

^b^Data missing from three manifest HDGECs at Time 1

^c^Data missing from one premanifest HDGEC at Time 0 and two manifest HDGECs at Time 1

^d^Data missing from two manifest HDGECs at Time 1

^e^Data missing from one manifest HDGEC at Time 0 and two manifest HDGECs at Time 1

To further examine the relationship between apathy and depression and to address potential overlap between measures, correlations between change scores for apathy and depression measures were calculated. Changes in the LARS Global score were significantly associated with changes in HAM-17 scores (*ρ* = 0.30, *p* = 0.030), but not with changes in clinician-rated depression on the PBA-s (*ρ* = 0.24, *p* = 0.086). In contrast, the two depression measures (HAM-17 and PBA-s Depression) were significantly associated with each other (*ρ* = 0.47, *p* < 0.001), and the two apathy measures (LARS Global score and PBA-s Apathy) were also significantly associated (*ρ* = 0.36, *p* = 0.007).

## Discussion

This longitudinal study examined the trajectories of apathy in a cohort of HDGECs over a 6-year period using a multidimensional apathy measure.

The results showed an overall increase in global apathy in HDGECs over time, driven primarily by worsening action initiation. Clinician-rated apathy on the PBA-s similarly increased over time, supporting a gradual reduction in goal-directed behavior. Within subgroups, premanifest participants exhibited a specific decline in action initiation, while manifest participants showed a general increase in global apathy. Finally, changes in depressive symptoms, but not other clinical measures, were correlated with changes in apathy over time.

These findings indicate that apathy severity shows a general increase in HDGECs over a 6-year period; however, the magnitude of change is small and highly variable. Individual trajectories differed substantially, with some participants demonstrating worsening and others showing stability or improvement. Notably, visual inspection of these trajectories suggests that variability was greatest among manifest HDGECs, who displayed both the largest increases and decreases in apathy severity over time.

The LARS is a self-report interview and therefore relies on intact insight and self-awareness, which may be reduced, particularly among manifest HDGECs. Although the LARS Self-Awareness subscale score showed a slight improvement over time in HDGECs (not significant after correction), this raises the possibility that changes in insight could contribute to both the observed variability in apathy trajectories and the small improvement in reported apathy in some participants. Apathy in HD has been linked to dysfunction in anterior cingulate–striatal circuits involved in motivation and goal-directed behavior, while unawareness is known to vary in HD and has been associated with broader frontostriatal dysfunction [36]. The overlap between these neural circuits may help explain why apathy and reduced awareness often co-occur in HD and may also contribute to discrepancies between self-reported and clinician-rated apathy as the disease progresses. This shared neural basis may therefore partly explain the variability observed in apathy progression across individuals. However, Mason and Barker [18] reported strong correspondence between participant and informant apathy ratings over 18 months, and previous work has shown that unawareness in HD is heterogeneous rather than uniformly impaired [37, 38]. Additionally, the significant association between change in LARS and PBA-s apathy in our study suggests that both self-report and clinician-rated measures capture similar longitudinal changes in apathy.

Together, these findings support the need for multimodal apathy assessment, including both self-report and clinician-rated measures, to diminish potential biases due to variable insight.

Across the full cohort of HDGECs there was a small average increase in apathy severity, with mean increases of 1.11 points on the PBA-s and 1.38 points on the LARS Global score, which was smaller than we expected over a 6-year period.

In line with these findings, Connors et al. [22] reported a small increase of approximately 0.5 points on the UHDRS behavioral apathy item over 5 years in the multinational COHORT study of 1082 manifest HDGECs, corresponding to an average increase of 0.1 points per year. In contrast, Thompson et al. [8] reported a substantially larger increase in apathy severity using the more comprehensive PBA-HD in a cohort of 111 manifest HDGECs followed for approximately 5 years, with longitudinal mixed-effects modeling estimating an average 0.88-point increase per year. These values are not directly comparable due to differences in scale structure and scoring range, but both studies suggest a gradual increase in apathy over time in manifest HD. This discrepancy in magnitude may reflect differences in measurement approach, disease stage, or cohort characteristics. The PBA-HD uses a smaller set of clinician-rated apathy items, whereas the LARS provides a broader multidimensional assessment, which may capture greater heterogeneity in apathy expression over time. Additionally, participants in the Thompson et al. [8] cohort were more functionally impaired according to TFC staging, which may partly account for the greater longitudinal increases observed.

Consistent with this interpretation, several longitudinal studies with shorter follow-up durations have not found significant changes in apathy severity over time. Mason and Barker [18] reported no significant change in self- or companion-rated apathy over approximately 18 months, and van Duijn et al. [20] similarly found stable apathy scores over a 2-year period in both premanifest and manifest HDGECs. Together, these findings suggest that longitudinal changes in apathy are generally small, variable, and difficult to detect over shorter observation periods, particularly when analyses focus on group averages rather than individual trajectories.

In premanifest participants, we observed a selective decline in Action Initiation apathy, extending our earlier cross-sectional work identifying this subtype in manifest HDGECs [15]. Previous studies in premanifest or early-stage HD such as Tabrizi et al. [11], have not found significant apathy progression, which may reflect shorter follow-up intervals and the limited sensitivity of unidimensional apathy measures. Most premanifest participants in the present study displayed worsening action initiation over 6 years, suggesting that impairments in self-initiated behavior may emerge early in the disease course. This selective decline may have a neurobiological basis, as early structural and functional changes in HD have been demonstrated in frontostriatal circuits, including the caudate and anterior cingulate cortex, which are critically involved in motivation and the initiation of goal-directed behavior [39, 40]. Consistent with this, De Paepe et al. [41] found that reduced middle cingulate cortex volume predicts increased apathy over time in HD, suggesting that early dysfunction in frontal–cingulate regions may contribute to the specific decline in action initiation observed in premanifest HDGECs. Although speculative, this pattern may reflect early disruption of frontostriatal circuits involved in goal-directed behavior.

By contrast, in manifest HDGECs, this selective impairment may broaden into more global apathy. This pattern is consistent with previous findings suggesting that apathy in earlier stages may be more closely linked to specific motivational and executive processes, whereas in later stages apathy becomes more multifactorial as motor, cognitive, and psychiatric symptoms accumulate and contribute to functional decline [35]. However, despite these differences in the pattern of subtype changes, the overall rate of apathy progression did not differ significantly between premanifest and manifest groups. This suggests that apathy progression may begin already in the premanifest stage and continue across disease progression, although the sample size may have limited statistical power to detect small differences between groups.

In the present study, longitudinal changes in apathy severity were modestly associated with changes in depressive symptoms, whereas changes in motor severity, cognition, and other markers of disease progression were not. However, the strength of this association was small, indicating that depression explained only a limited proportion of variance in apathy change. Changes in motor symptoms were instead correlated with changes in cognition, executive functioning, and social cognition, but not with changes in apathy, suggesting that apathy progression may be heterogeneous and less tightly linked to disease progression than many other clinical features. Previous studies have largely examined apathy cross-sectionally or evaluated baseline apathy as a predictor of later functional or clinical outcomes, rather than assessing within-person changes over time [10, 11, 19].

While cross-sectional studies consistently report associations between apathy severity and clinical variables such as motor impairment, depressive symptoms, and cognitive dysfunction [15], the present findings indicate that longitudinal changes in apathy are only slightly related to changes in depressive symptoms. This association should be interpreted with caution, as some items on depression scales such as the HAM-17 may overlap with apathy-related symptoms (e.g., reduced activity and psychomotor slowing). However, changes in clinician-rated depression on the PBA-s were not significantly associated with changes in apathy in the present study, whereas the depression measures were associated with each other and the apathy measures were associated with each other, suggesting that apathy and depression are related but partially distinct syndromes rather than reflecting a single construct measured differently. Importantly, the small effect size suggests that depressive symptom change accounts for only a small proportion of apathy progression, supporting the idea that apathy represents a partially independent neuropsychiatric feature in HD.

To our knowledge, this is the first study to examine apathy longitudinally for a period exceeding 5 years using a multidimensional measure. Nevertheless, some limitations must be considered.

As in most longitudinal studies, some participants were lost to follow-up. Those who did not return had poorer baseline cognitive performance, more severe motor symptoms, and greater disease burden than those that were reevaluated. This selective dropout may have biased the follow-up sample toward individuals with less severe apathy progression. The study sample comprised HDGECs who were willing and able to participate in long-term follow-up. Consequently, individuals with more advanced disease, greater motor impairment, or more severe cognitive deficits are likely underrepresented, and the observed patterns of apathy progression may not be fully representative of all individuals with HD.

Notably, however, baseline apathy did not differ between groups, which supports the interpretation that apathy may follow a more heterogeneous and less severity-dependent trajectory. Variability in individual apathy trajectories, particularly among manifest HDGECs, may also reflect unmeasured factors such as major life events, changes in pharmacological treatment or management of psychiatric symptoms, which were not systematically assessed in the present study. In addition, the relatively long interval between assessments (approximately 6 years) means that shorter-term fluctuations or non-linear patterns of change in apathy may not have been captured, and the net change observed may mask more complex trajectories over time. Future studies should further examine apathy progression using designs that allow for repeated multidimensional assessments over time and incorporate information on treatment exposure and psychiatric management to better account for individual variability.

An additional limitation relates to the classification of premanifest and manifest participants. There is considerable variability in how disease stage is defined across HD studies, including classification based on TFC scores, motor symptom severity, or more recent biological staging frameworks [42]. In the present study, classification was based on a UHDRS-TMS cutoff previously applied in our cohort, which represents a relatively conservative threshold for defining manifest HD. Differences in staging criteria across studies may, therefore, influence comparisons of group-level findings, as the categorization of individuals as premanifest or manifest may vary depending on the criteria used. Simultaneously, the use of a low motor threshold may allow detection of changes emerging during very early or prodromal stages of HD. Future studies may benefit from harmonizing staging definitions to improve comparability.

Although the sample size is comparable to other longitudinal HD studies [8, 14, 18] and substantial for a 6-year follow-up, the detection of small associations between changes in apathy and changes in other clinical variables may still be limited. While the use of change scores (Δ values) is intuitive and clinically interpretable, they may not fully capture subtle or variable patterns of change, especially in the context of high individual variation.

Finally, it is also important to consider whether current measures fully capture the construct of apathy in HD. Although apathy is widely reported across neurodegenerative diseases, there is limited consensus regarding its definition and phenomenology, and it may manifest differently across diseases and clinical stages [43]. In HD, apathy is frequently reported by caregivers rather than by individuals themselves, and individuals with more pronounced apathy may experience little subjective distress or reduced awareness of motivational change. As a result, measured apathy may partly reflect awareness and subjective experience rather than motivational impairment alone. These challenges highlight the need for complementary approaches to apathy assessment including qualitative work exploring how individuals with HD experience apathy, to better understand and assess this construct in HD.

## Conclusion

In conclusion, this six-year longitudinal study demonstrates that apathy in HDGECs increases modestly over time but follows highly variable individual trajectories. Action Initiation emerged as the earliest and most consistently changing apathy domain, particularly in premanifest participants, suggesting that motivational difficulties related to initiation of behavior may arise before broader apathy becomes apparent. In manifest HDGECs, apathy changes were more heterogeneous and extended beyond specific subdomains, consistent with increasing clinical complexity and inter-individual variability at later disease stages. Changes in depressive symptoms showed a small correlation with changes in apathy, whereas changes in other clinical measures did not, supporting the view that apathy represents a largely independent and variable neuropsychiatric feature of HD. Together, these findings highlight the importance of long-term follow-up and multidimensional assessment when studying apathy progression and underscore the need to account for individual variability in both research and clinical practice.

## Data Availability

The data from this study are available upon reasonable request from the corresponding author. The data are not publicly available due to privacy or ethical restrictions.

## References

[1] The Huntington’s Disease Collaborative Research Group (1993) A novel gene containing a trinucleotide repeat that is expanded and unstable on Huntington’s disease chromosomes. Cell 72:971–983. 10.1016/0092-8674(93)90585-e 8458085 10.1016/0092-8674(93)90585-e

[2] Bates GP, Dorsey R, Gusella JF et al (2015) Huntington disease. Nat Rev Dis Primers 1:15005. 10.1038/nrdp.2015.5 27188817 10.1038/nrdp.2015.5

[3] McColgan P, Tabrizi SJ (2018) Huntington’s disease: a clinical review. Eur J Neurol 25:24–34. 10.1111/ene.13413 28817209 10.1111/ene.13413

[4] Duff K, Paulsen J, Mills J et al (2010) Mild cognitive impairment in prediagnosed Huntington disease. Neurology 75:500–507. 10.1212/WNL.0b013e3181eccfa2 20610833 10.1212/WNL.0b013e3181eccfa2 PMC2918475

[5] Matmati J, Verny C, Allain P (2022) Apathy and Huntington’s disease: a literature review based on PRISMA. J Neuropsychiatry Clin Neurosci 34:100–112. 10.1176/appi.neuropsych.21060154 34961332 10.1176/appi.neuropsych.21060154

[6] Paulsen JS, Miller AC, Hayes T, Shaw E (2017) Cognitive and behavioral changes in Huntington disease before diagnosis. Handb Clin Neurol 144:69–91. 10.1016/B978-0-12-801893-4.00006-7 28947127 10.1016/B978-0-12-801893-4.00006-7

[7] Paulsen JS (2011) Cognitive impairment in Huntington disease: diagnosis and treatment. Curr Neurol Neurosci Rep 11:474–483. 10.1007/s11910-011-0215-x 21861097 10.1007/s11910-011-0215-x PMC3628771

[8] Thompson JC, Harris J, Sollom AC et al (2012) Longitudinal evaluation of neuropsychiatric symptoms in Huntington’s disease. J Neuropsychiatry Clin Neurosci 24:53–60. 10.1176/appi.neuropsych.11030057 22450614 10.1176/appi.neuropsych.11030057

[9] Camacho M, Barker RA, Mason SL (2018) Apathy in Huntington’s disease: a review of the current conceptualization. J Alzheimers Dis Parkinsonism. 10.4172/2161-0460.1000431

[10] Ruiz-Idiago J, Pomarol-Clotet E, Salvador R (2023) Longitudinal analysis of neuropsychiatric symptoms in a large cohort of early-moderate manifest Huntington’s disease patients. Parkinsonism Relat Disord 106:105228. 10.1016/j.parkreldis.2022.11.020 36470173 10.1016/j.parkreldis.2022.11.020

[11] Tabrizi SJ, Scahill RI, Owen G et al (2013) Predictors of phenotypic progression and disease onset in premanifest and early-stage Huntington’s disease in the TRACK-HD study: analysis of 36-month observational data. Lancet Neurol 12:637–649. 10.1016/S1474-4422(13)70088-7 23664844 10.1016/S1474-4422(13)70088-7

[12] Marin RS (1990) Differential diagnosis and classification of apathy. Am J Psychiatry 147:22–30. 10.1176/ajp.147.1.22 2403472 10.1176/ajp.147.1.22

[13] Levy R, Dubois B (2006) Apathy and the functional anatomy of the prefrontal cortex–basal ganglia circuits. Cereb Cortex 16:916–928. 10.1093/cercor/bhj043 16207933 10.1093/cercor/bhj043

[14] De Paepe AE, Garcia-Gorro C, Martinez-Horta S et al (2022) Delineating apathy profiles in Huntington’s disease with the short-Lille Apathy Rating Scale. Parkinsonism Relat Disord 105:83–89. 10.1016/j.parkreldis.2022.10.025 36395542 10.1016/j.parkreldis.2022.10.025

[15] Hendel RK, Hellem MNN, Hjermind LE et al (2021) Intellectual curiosity and action initiation are subtypes of apathy affected in Huntington disease gene expansion carriers. Cogn Behav Neurol 34:295–302. 10.1097/WNN.0000000000000286 34851867 10.1097/WNN.0000000000000286

[16] Andrews SC, Langbehn DR, Craufurd D et al (2021) Apathy predicts rate of cognitive decline over 24 months in premanifest Huntington’s disease. Psychol Med 51:1338–1344. 10.1017/S0033291720000094 32063235 10.1017/S0033291720000094

[17] Hare E, Bachoud-Lévi A-C, Reilmann R et al (2022) Cognitive processes of apathy in Huntington’s disease show high sensitivity to disease progression. Clin Park Relat Disord 7:100168. 10.1016/j.prdoa.2022.100168 36405870 10.1016/j.prdoa.2022.100168 PMC9673112

[18] Mason S, Barker RA (2015) Rating apathy in Huntington’s disease: patients and companions agree. J Huntingtons Dis 4:49–59 26333257

[19] Reedeker N, Bouwens JA, van Duijn E et al (2011) Incidence, course, and predictors of apathy in Huntington’s disease: a two-year prospective study. J Neuropsychiatry Clin Neurosci 23:434–441. 10.1176/jnp.23.4.jnp434 22231315 10.1176/jnp.23.4.jnp434

[20] van Duijn E, Reedeker N, Giltay EJ et al (2014) Course of irritability, depression and apathy in Huntington’s disease in relation to motor symptoms during a two-year follow-up period. Neurodegener Dis 13:9–16. 10.1159/000343210 23948661 10.1159/000343210

[21] The Huntington Study Group COHORT Investigators (2012) Characterization of a large group of individuals with Huntington disease and their relatives enrolled in the COHORT study. PLoS ONE 7:e29522. 10.1371/journal.pone.0029522 22359536 10.1371/journal.pone.0029522 PMC3281013

[22] Connors MH, Teixeira-Pinto A, Loy CT (2023) Apathy and depression in Huntington’s disease: distinct longitudinal trajectories and clinical correlates. J Neuropsychiatry Clin Neurosci 35:69–76. 10.1176/appi.neuropsych.21070191 36128678 10.1176/appi.neuropsych.21070191

[23] Baake V, Coppen EM, van Duijn E et al (2018) Apathy and atrophy of subcortical brain structures in Huntington’s disease: a two-year follow-up study. Neuroimage Clin 19:66–70. 10.1016/j.nicl.2018.03.033 30035003 10.1016/j.nicl.2018.03.033 PMC6051315

[24] Callaghan J, Stopford C, Arran N et al (2015) Reliability and factor structure of the Short Problem Behaviors Assessment for Huntington’s disease (PBA-s) in the TRACK-HD and REGISTRY studies. J Neuropsychiatry Clin Neurosci 27:59–64. 10.1176/appi.neuropsych.13070169 25716488 10.1176/appi.neuropsych.13070169

[25] Hasselbalch SG, Andersen BB, Ejlerskov P et al (2025) The Danish Dementia Research Centre: integrating patient care, clinical research, and national educational services. J Alzheimer’s Dis. 10.1177/13872877251365195 10.1177/13872877251365195 40776623

[26] Huntington Study Group (1996) Unified Huntington’s disease rating scale: reliability and consistency. Huntington Study Group. Mov Disord 11:136–142. 10.1002/mds.870110204 8684382 10.1002/mds.870110204

[27] Folstein MF, Folstein SE, McHugh PR (1975) Mini-Mental State. J Psychiatr Res 12:189–198. 10.1016/0022-3956(75)90026-6 1202204 10.1016/0022-3956(75)90026-6

[28] Nasreddine ZS, Phillips NA, Bédirian V et al (2005) The Montreal Cognitive Assessment, MoCA: a brief screening tool for mild cognitive impairment. J Am Geriatr Soc 53:695–699. 10.1111/j.1532-5415.2005.53221.x 15817019 10.1111/j.1532-5415.2005.53221.x

[29] Vinther-Jensen T, Larsen IU, Hjermind LE et al (2014) A clinical classification acknowledging neuropsychiatric and cognitive impairment in Huntington’s disease. Orphanet J Rare Dis. 10.1186/s13023-014-0114-8 25026978 10.1186/s13023-014-0114-8 PMC4105878

[30] Hamilton M (1960) A rating scale for depression. J Neurol Neurosurg Psychiatry 23:56–62. 10.1136/jnnp.23.1.56 14399272 10.1136/jnnp.23.1.56 PMC495331

[31] Sockeel P (2006) The Lille apathy rating scale (LARS), a new instrument for detecting and quantifying apathy: validation in Parkinson’s disease. J Neurol Neurosurg Psychiatry 77:579–584. 10.1136/jnnp.2005.075929 16614016 10.1136/jnnp.2005.075929 PMC2117430

[32] Craufurd D, Thompson JC, Snowden JS (2001) Behavioral changes in Huntington disease. Neuropsychiatry Neuropsychol Behav Neurol 14:219–226 11725215

[33] Smith A (1982) Symbol digit modalities test (SDMT). Western Psychological Services, Los Angeles

[34] Calder AJ (1996) Facial emotion recognition after bilateral amygdala damage: differentially severe impairment of fear. Cogn Neuropsychol 13:699–745. 10.1080/026432996381890

[35] Hendel RK, Hellem MNN, Hjermind LE et al (2023) On the association between apathy and deficits of social cognition and executive functions in Huntington’s disease. J Int Neuropsychol Soc 29:369–376. 10.1017/S1355617722000364 36189712 10.1017/S1355617722000364

[36] McCusker E, Loy CT (2014) The many facets of unawareness in Huntington disease. Tremor Other Hyperkinet Mov 4:257. 10.5334/tohm.231 10.7916/D8FJ2FD3 PMC4231168 25411649

[37] Caine ED, Shoulson I (1983) Psychiatric syndromes in Huntington’s disease. Am J Psychiatry 140:728–733. 10.1176/ajp.140.6.728 6221669 10.1176/ajp.140.6.728

[38] Hoth KF, Paulsen JS, Moser DJ et al (2007) Patients with Huntington’s disease have impaired awareness of cognitive, emotional, and functional abilities. J Clin Exp Neuropsychol 29:365–376. 10.1080/13803390600718958 17497560 10.1080/13803390600718958

[39] Montoya A, Price BH, Menear M, Lepage M (2006) Brain imaging and cognitive dysfunctions in Huntington’s disease. J Psychiatry Neurosci 31:21–29 16496032 PMC1325063

[40] Reading SAJ, Dziorny AC, Peroutka LA et al (2004) Functional brain changes in presymptomatic Huntington’s disease. Ann Neurol 55:879–883. 10.1002/ana.20121 15174024 10.1002/ana.20121

[41] De Paepe AE, Ara A, Garcia-Gorro C et al (2021) Gray matter vulnerabilities predict longitudinal development of apathy in Huntington’s disease. Mov Disord 36:2162–2172. 10.1002/mds.28638 33998063 10.1002/mds.28638

[42] Tabrizi SJ, Schobel S, Gantman EC et al (2022) A biological classification of Huntington’s disease: the integrated staging system. Lancet Neurol 21:632–644. 10.1016/S1474-4422(22)00120-X 35716693 10.1016/S1474-4422(22)00120-X

[43] Ishii S, Weintraub N, Mervis JR (2009) Apathy: a common psychiatric syndrome in the elderly. J Am Med Dir Assoc 10:381–393. 10.1016/j.jamda.2009.03.007 19560715 10.1016/j.jamda.2009.03.007

